# Leucettinib-21 decreases dosage effects of *DYRK1A* in human trisomy 21 induced pluripotent stem cell-derived neural cells

**DOI:** 10.1242/dmm.052740

**Published:** 2026-06-29

**Authors:** Nicole R. West, Mattias F. Lindberg, Julien Dairou, Shawn MacGregor, Sahith Puthireddy, Laurent Meijer, Anita Bhattacharyya

**Affiliations:** ^1^ Waisman Center, University of Wisconsin-Madison, Madison, WI, USA; ^2^ Cellular and Molecular Biology Graduate Program, University of Wisconsin-Madison, Madison WI 53706, USA; ^3^ Perha Pharmaceuticals, Perharidy Peninsula, 29680 Roscoff, France; ^4^ Laboratoire de Chimie et Biochimie Pharmacologiques et Toxicologiques, UMR 8601 CNRS, Université Paris Cité, Paris 75270, France; ^5^ Department of Cell and Regenerative Biology, School of Medicine and Public Health, University of Wisconsin-Madison, Madison, WI 53705, USA

**Keywords:** DYRK1A, DYRK1A inhibitors, Down syndrome, Induced pluripotent stem cells, Cell proliferation, Tau phosphorylation

## Abstract

Dosage imbalance of dual specificity tyrosine phosphorylation regulated kinase 1A (*DYRK1A*) is a feature of several neurodevelopmental and neurodegenerative diseases, including Down syndrome, DYRK1A syndrome, autism spectrum disorders, Alzheimer's disease and Parkinson's disease. Thus, manipulating DYRK1A activity in the brain has emerged as a potential therapeutic target for neurological disorders. Several DYRK1A inhibitors have shown promise for improving cognition in rodent models of Down syndrome and Alzheimer's disease, for example, but the ability of these inhibitors to affect DYRK1A levels or activity in relevant human cells has not been established. We filled this gap by testing the effects of a new DYRK1A inhibitor on trisomy 21 induced pluripotent stem cell (iPSC)-derived neural progenitor cells and neurons, in which DYRK1A expression and activity are increased. Our results demonstrated that Leucettinib-21, a potent and selective low-molecular-mass pharmacological inhibitor of DYRK1A, decreases DYRK1A activity in human trisomy 21 iPSC-derived neural progenitor cells and cortical neurons. Leucettinib-21 reduces DYRK1A activity in a relevant human disease model, supporting future human trials.

## Teaser

**Summary:** We show, for the first time, that Leucettinib-21, a pharmacological inhibitor of DYRK1A, decreases DYRK1A kinase activity in a human induced pluripotent stem cell-derived neural cell culture model of Down syndrome.

## INTRODUCTION

Dual specificity tyrosine phosphorylation regulated kinase 1A (DYRK1A) is evolutionarily conserved and belongs to the CMGC family of kinases that play a role in a wide range of cellular functions (Kannan and Neuwald, 2004; Aranda et al., 2011; Soundararajan et al., 2013; Hämmerle et al., 2008). DYRK1A autophosphorylates a tyrosine residue within its activation loop and phosphorylates serine and threonine residues on its substrates (Duchon and Herault, 2016; Atas-Ozcan et al., 2021; Rammohan et al., 2022; Soundararajan et al., 2013; Becker and Joost, 1998). DYRK1A is ubiquitously expressed in organs across development and adulthood and plays a key role in maintaining normal cell proliferation, differentiation and function (Atas-Ozcan et al., 2021; Guimera et al., 1999; Deboever et al., 2022; Becker and Joost, 1998; Hämmerle et al., 2008). *DYRK1A* is a dosage-sensitive gene, and its dosage imbalance has been linked to several neurological disorders, diabetes, cancer and heart disease (Deboever et al., 2022).

### *DYRK1A* is dosage sensitive

Proper gene dosage of *DYRK1A* is necessary for normal brain development and function, and disrupting this balance causes neurological disorders. DYRK1A syndrome, a rare *DYRK1A* haploinsufficiency, is among the most common monogenic forms of intellectual disability (Courraud et al., 2021). DYRK1A syndrome results from microdeletions in chromosome 21q22.12q22.3, single-nucleotide variants, translocations, or small insertions or deletions in the gene (Ji et al., 2015). Microcephaly, intellectual disability, autism spectrum disorder, speech impairment, seizures, skeletal and gait abnormalities, and eye defects are characteristic features of DYRK1A syndrome (Ji et al., 2015).

DYRK1A overexpression has been linked to several cancers, including acute leukemia, glioblastoma, head and neck squamous cell carcinoma, hepatocellular carcinoma and pancreatic ductal adenocarcinoma owing to its role in cell cycle progression, DNA damage repair and cancer stem cell maintenance (Rammohan et al., 2022; Fernández-Martínez et al., 2015; Boni et al., 2020). DYRK1A promotes tumor growth in some cellular contexts, and DYRK1A overexpression correlates with poor prognosis in certain cancers (Laham et al., 2022, 2024; Li et al., 2019).

*DYRK1A* is encoded on human chromosome 21 (Hsa21) and is overexpressed in Down syndrome [DS; trisomy 21 (T21)] (Lejeune et al., 1959). DS is the leading genetic cause of intellectual disability, with IQs in individuals with DS ranging from mild to severe and an average IQ being around 50 (Mégarbané et al., 2013; Kłosowska et al., 2022; Lejeune et al., 1959; Hassold and Hunt, 2001; Fidler and Nadel, 2007; Hattori et al., 2000), accompanied by structural deficits in the brain. In addition to the neurological symptoms, individuals with DS have increased risk of congenital heart defects, leukemia, gastrointestinal problems, vision and hearing impairment, and early onset of Alzheimer's disease (AD) (Roizen, 2010; Capone et al., 2018; Lott and Head, 2019). The role of DYRK1A overexpression in the cellular processes that regulate these diverse organ defects has been an area of intense research and potential therapeutics.

### Manipulating DYRK1A expression and activity

The critical role of DYRK1A in cell proliferation, differentiation of neurons and synaptic plasticity has made it a therapeutic target for cancer, diabetes and neurological disorders, including DS and AD (Rammohan et al., 2022; Barzowska et al., 2021; Deboever et al., 2022; Arbones et al., 2019; Duchon and Herault, 2016; Abbassi et al., 2015). Several DYRK1A inhibitors exist, including Epigallocatechin gallate (EGCG), Harmine, Leucettine L41, Leucettinib-21 (LCTB-21), Lorecivivint, Cirtuvivint, PST-001 and others (Lindberg et al., 2023a,b). Compound screens reveal Leucettinibs as a potent class of DYRK1A kinase inhibitors (Deau et al., 2023; Lindberg et al., 2023a,b; Lindberg and Meijer, 2021). Notably, LCTB-21 is the only DYRK1A inhibitor that has shown efficacy in animal models of DS and has progressed to clinical trials (Meijer et al., 2024), highlighting the need for thorough evaluation of LCTB-21 in human cells. The goal of this study was to determine whether LCTB-21 is able to inhibit DYRK1A activity and, consequently, reduce the phosphorylation of downstream targets in human cells.

LCTB-21 – a potent, low-molecular-mass DYRK1A inhibitor developed by Perha Pharmaceuticals – improves memory, learning and spatial recognition in mouse models of DS and AD (Lindberg et al., 2023b; Deau et al., 2023) and has passed preclinical regulatory safety trials in rats and minipigs (Meijer et al., 2024). Twenty-eight-day toxicology studies across varying doses determined a no observed adverse effect level of 20 mg/kg for LCTB-21 in rats and 100 mg/kg in minipigs (Meijer et al., 2024). Notably, LCTB-21 effectively corrects cognitive disorders in animal models at much lower doses (0.3–0.5 mg/kg) (Meijer et al., 2024).

Although mouse models have been integral to understanding cellular and molecular mechanisms of DS (Herault et al., 2017; Rueda et al., 2012), Hsa21 is orthologous to sections of mouse chromosomes 10, 16 and 17 (Mmu10, Mmu16 and Mmu17, respectively), so these models do not fully recapitulate the molecular or clinical features of people with DS (Xing et al., 2016; Granholm, 2025; Gardiner et al., 2003; Bales, 2012). More broadly, although therapeutics often show promise in rodent models, most fail to show efficacy in human clinical trials (Nikanjam et al., 2022; Mak et al., 2014; Kim et al., 2022; Oxford et al., 2020), highlighting a need for human models to screen drug candidates and delineate mechanisms of action.

LCTB-21 is currently in a Phase 1 clinical trial to investigate its pharmacokinetics, safety and tolerability in healthy volunteers, followed by evaluation in a small cohort of individuals with DS and patients with AD (NCT06206824) (Meijer et al., 2024). However, this drug candidate has not yet been evaluated in a human model of DS, which could provide insight into the mechanism of action of LCTB-21.

We tested the efficacy of LCTB-21 in human T21 induced pluripotent stem cell (iPSC)-derived neural cells, a human model of DS. The kinase-inactive isomer iso-Leucettinib-21 (iso-LCTB-21) was used as a negative control. Our results demonstrate that LCTB-21 reduces DYRK1A activity and subsequent phosphorylation of DYRK1A targets in human iPSC-derived neural cells. LCTB-21 may represent a promising drug candidate for inhibiting DYRK1A and, ultimately, relieving cell cycle deficits and neuronal dysfunction in DS and other neurological diseases. This work also establishes a disease-relevant cellular model that can be used in future studies to delineate the mechanism of action of LCTB-21.

## RESULTS

### LCTB-21 inhibits the activity of DYRK1A in human iPSC-derived neural progenitor cells

**Fig. 1.**
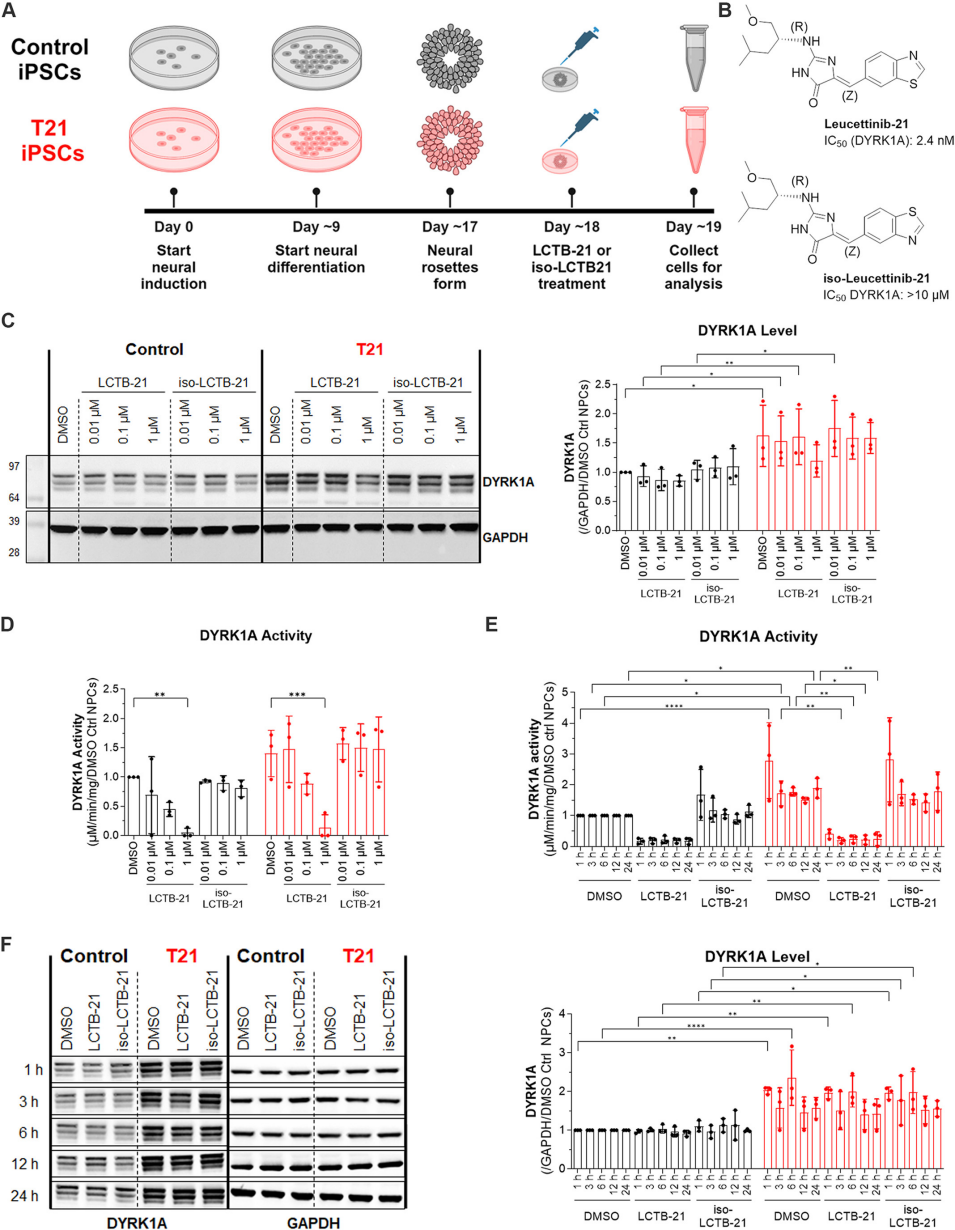
**Leucettinib-21 reduces the activity of DYRK1A in human induced pluripotent stem cell-derived control and trisomy 21 neural progenitor cells.** (A) Schematic of induced pluripotent stem cell (iPSC)-derived neural progenitor cell (NPC) differentiation and treatment. Created in BioRender by Bhattacharyya, A. (2026). https://BioRender.com/j8to9gm. This figure was sublicensed under CC-BY 4.0 terms. (B) Structure of Leucettinib-21 (LCTB-21) and its kinase-inactive isomer, iso-Leucettinib-21 (iso-LCTB-21). Half-maximal inhibitory concentration (IC_50_) values on DYRK1A are provided. (C) Western blot and quantification of DYRK1A levels in control and trisomy 21 (T21) NPCs (*n*=3). DYRK1A protein is increased in T21 NPCs, and treatment with LCTB-21 does not affect the protein levels of DYRK1A. The same GAPDH control is shared with Fig. 2B as multiple targets were probed simultaneously on the membrane. (D) Quantification of DYRK1A activity in NPCs (*n*=3). DYRK1A activity is increased in T21 NPCs. DYRK1A activity is decreased in a dose-dependent manner in control and T21 NPCs when treated with increasing concentrations of LCTB-21. (E) Quantification of DYRK1A activity in NPCs over 24 h (*n*=3). DYRK1A activity of DMSO-treated T21 NPCs is increased relative to that in DMSO-treated control NPCs at the respective times. DYRK1A activity in T21 NPCs is decreased within 1 h and persists for at least 24 h when treated with 1 μM LCTB-21 compared to that in DMSO-treated T21 NPCs at the respective times. (F) Western blot and quantification of DYRK1A levels in control and T21 NPCs over time (*n*=3). DYRK1A is increased in T21 NPCs compared to that in control NPCs at the respective times and treatment conditions. Treatment with LCTB-21 does not affect the levels of DYRK1A. The same GAPDH control is shared with Fig. 2F as multiple targets were probed simultaneously on the membrane. Results are from at least three technical replicates from separate stem cell differentiations (batches). The sample size (*n*) is defined by the number of batches. Data are presented as mean±s.d. *P*-values from two-way ANOVA with post-hoc tests are presented as *P≤0.05, **P≤0.01, ***P≤0.001, ****P≤0.0001. Two-way ANOVA results, post-hoc tests, *P*-values and excluded outliers are listed in Table S3.

Because DYRK1A plays a role in regulating cell cycle progression, we tested the efficacy of LCTB-21 in proliferating neural progenitor cells (NPCs). NPCs were differentiated from T21 (DS) and isogenic control (euploid) iPSCs (Fig. 1A) (Hunt et al., 2019; Fedorova et al., 2019) and treated with DMSO, LCTB-21 or iso-LCTB-21 (Fig. 1B). There was no difference in cell survival or DYRK1A activity in NPCs treated with DMSO compared to that in untreated control NPCs (Fig. S1A), so LCTB-21 and iso-LCTB-21 treatments were compared to the DMSO vehicle in subsequent experiments. Control and T21 cultures had similarly enriched proportions of PAX6^+^ and SOX2^+^ NPCs, and treatment with LCTB-21 or iso-LCTB-21 did not affect the cellular composition compared to that with the DMSO vehicle (Fig. S1B–D). As previously reported (Martí et al., 2003), DYRK1A protein is expressed in the cytoplasm and the nucleus and is localized similarly in control and T21 NPCs (Fig. S1E).

**Fig. 2.**
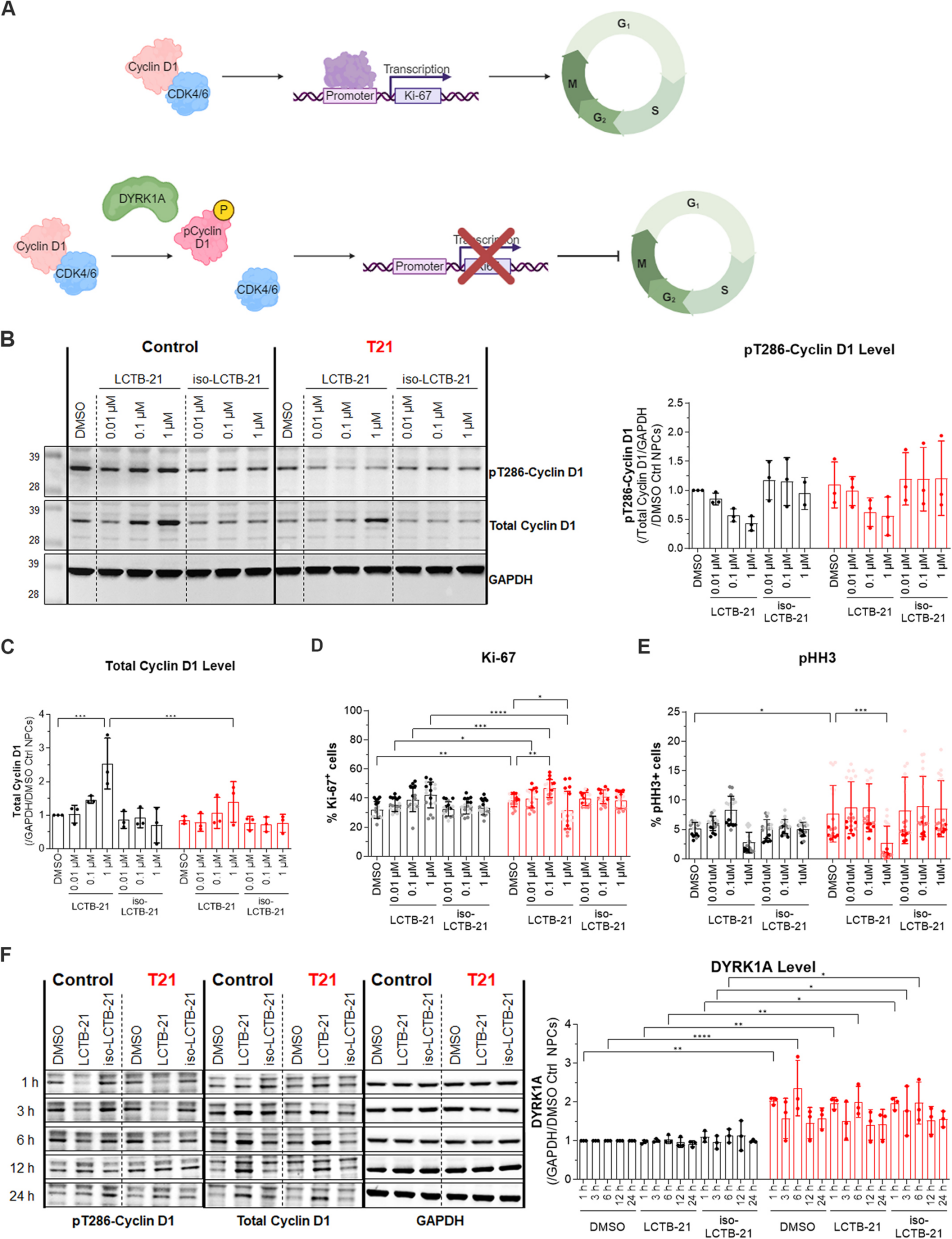
**DYRK1A inhibition decreases phosphorylation of the DYRK1A target, cyclin D1, in human iPSC-derived neural progenitor cells.** (A) Schematic of DYRK1A and cyclin D1 phosphorylation. When cyclin D1 is phosphorylated by DYRK1A, downstream cell cycle genes, including *MKI67* (encoding Ki-67), are not transcribed. Created in BioRender by Bhattacharyya, A. (2026). https://BioRender.com/j34f58i. This figure was sublicensed under CC-BY 4.0 terms. (B) Western blot and quantification of cyclin D1 and pT286-cyclin D1 levels in control and T21 NPCs (*n*=3). pT286-cyclin D1 levels decrease as DYRK1A activity decreases with increasing doses of LCTB-21. The same GAPDH control is shared with Fig. 1C as multiple targets were probed simultaneously on the membrane. (C) Cyclin D1 levels increase with increasing doses of LCTB-21 (*n*=3). (D) Ki-67^+^ cells in the cell cycle. More T21 NPCs are Ki-67^+^, and, with the exception of T21 NPCs treated with 1 μM LCTB-21, the number of Ki-67^+^ cells increase with LCTB-21 treatment (*n*=3). (E) pHH3^+^ cells in late G2/M phases. Fewer NPCs are in late G2/M phases with 1 μM LCTB-21 treatment (*n*=3). (F) Western blot and quantification of cyclin D1 and pT286-cyclin D1 levels in control and T21 NPCs over time (*n*=3). pT286-cyclin D1 is decreased in control and T21 NPCs within 1 h and persists for at least 24 h when treated with 1 μM LCTB-21 relative to that in respective DMSO-treated NPCs. The same GAPDH control is shared with Fig. 1F as multiple targets were probed simultaneously on the membrane. Results are from at least three technical replicates from separate stem cell differentiations (batches). The sample size (*n*) is defined by the number of batches. Data are presented as mean±s.d. *P*-values from two-way ANOVA with post-hoc tests are presented as *P≤0.05, **P≤0.01, ***P≤0.001, ****P≤0.0001. Two-way ANOVA results, post-hoc tests, *P*-values and excluded outliers are listed in Table S3.

To assess the cytotoxicity of LCTB-21 and iso-LCTB-21 in iPSC-derived NPCs, cell viability was assessed with the CellTiter-Glo assay, which measures intracellular ATP levels. Averaged across three technical replicates, ATP levels in LCTB-21-treated control and trisomic NPCs were 93% and 88% of the levels in their respective DMSO-treated controls (Fig. S1F). According to the International Organization for Standardization (ISO) criteria, which define anything reducing cell viability by more than 30% as cytotoxic (ISO Standards, 2022; Gruber and Nickel, 2023), 1 μM LCTB-21 is not cytotoxic to either control or T21 iPSC-derived NPCs, although a slight reduction in viability was still observed.

We assessed the expression of DYRK1A protein in iPSC-derived NPCs. As expected, DYRK1A protein levels were increased in T21 NPCs relative to those in control NPCs (Fig. 1C). Protein levels of DYRK1A did not change significantly in control or T21 NPCs following the addition of LCTB-21 or iso-LCTB-21 compared to those following the addition of the DMSO control. However, there was a slight decrease in the DYRK1A levels in T21 NPCs treated with 1 μM LCTB-21 (Fig. 1C). LCTB-21 inhibition of DYRK1A activity at the 1 μM dose may prevent DYRK1A autophosphorylation at Ser97, leading to its degradation (Kii et al., 2016).

DYRK1A kinase activity was increased ∼1.5-fold in T21 NPCs compared to that in control NPCs. Treatment with LCTB-21 decreased DYRK1A activity in a dose-dependent manner in both control and T21 NPCs, consistent with observations in mouse HT-22 hippocampal cells (Lindberg et al., 2023b) (Fig. 1D). To determine the time course of LCTB-21 action on DYRK1A kinase activity, DYRK1A kinase activity was measured in NPCs treated with 1 μM LCTB-21 at 1, 3, 6, 12 and 24 h (Fig. 1E). DYRK1A activity was reduced by more than 80% in control and T21 NPCs within 1 h of LCTB-21 treatment, and reduction of DYRK1A activity persisted for at least 24 h after LCTB-21 treatment. Consistent with the dose–response assay (Fig. 1C), increased DYRK1A protein level was detected in T21 NPCs. LCTB-21 and iso-LCTB-21 treatments did not affect DYRK1A protein levels (Fig. 1F). These results demonstrated that LCTB-21 is effective at inhibiting the activity of DYRK1A protein in human iPSC-derived NPCs while not affecting its expression.

### DYRK1A inhibition decreases phosphorylation of the DYRK1A target, cyclin D1, in human iPSC-derived NPCs

Cyclin D1 is phosphorylated by DYRK1A at Thr286 (pT286-cyclin D1) (Soppa et al., 2014; Ashford et al., 2014) and plays an integral role in the expression of cell cycle genes, including *MKI67* (encoding Ki-67) (Hille et al., 2016), that regulate cell cycle progression (Rammohan et al., 2022; Chen et al., 2013; Laham et al., 2024). DYRK1A imbalance leads to altered cell cycle progression in mouse and cell models, with DYRK1A overexpression inhibiting cell proliferation (Chakrabarti et al., 2007; Sharma et al., 2022; Najas et al., 2015; Yabut et al., 2010; Hämmerle et al., 2011; Park et al., 2010) (Fig. 2A). Because LCTB-21 reduced the activity of DYRK1A in NPCs, we asked whether LCTB-21 has downstream effects on the phosphorylation of the DYRK1A target, cyclin D1.

We found a dose-dependent reduction in the phosphorylation of cyclin D1 at Thr286 relative to total cyclin D1 in both control and T21 NPCs when treated with LCTB-21 (Fig. 2B), concomitant with the dose-dependent decrease in DYRK1A activity (Fig. 1D). Thr286-phosphorylated cyclin D1 is unstable, and dephosphorylated cyclin D1 shows increased stability (Diehl et al., 1997). DYRK1A inhibition by LCTB-21 thus led to cyclin D1 accumulation in control and T21 NPCs (Fig. 2C).

We next asked whether reduction of DYRK1A activity alters the proliferation of NPCs. We analyzed the number of cycling (Ki-67^+^) cells upon LCTB-21 treatment (Fig. 2D). More T21 NPCs were cycling upon LCTB-21 treatment relative to control NPCs (Fig. 2D). The number of Ki-67^+^ NPCs slightly increased in proportion to LCTB-21 dosage in control cells. The trend was the same in T21 NPCs, with the exception of the 1 μM dose, at which the decrease in T21 Ki-67^+^ cells was likely due to toxicity (Fig. S1F). DYRK1A inhibition led to a reduction in pT286-cyclin D1 and the accumulation of cyclin D1, with a concomitant increase in Ki-67^+^ NPCs in the cell cycle. Cyclin D1 levels are high during G­_1_, and cyclin D1 levels are reduced as the cell progresses to S phase (Yang et al., 2006), typically through the phosphorylation of cyclin D1 at Thr286 and subsequent degradation (Diehl et al., 1997). The accumulation of cyclin D1 and decreased levels of pT286-cyclin D1 (Fig. 2B,C) raised the possibility that Ki-67^+^ control and T21 NPCs (Fig. 2D) accumulate in the G_1_ phase and do not progress to S phase when treated with LCTB-21. To test this, we analyzed the number of cells positive for phosphohistone H3 (pHH3), a marker for late G_2_ and M phases. Consistent with the Ki-67 data, there were more pHH3^+^ T21 NPCs than pHH3^+^ control NPCs (Fig. 2E). Fewer control and T21 NPCs were pHH3^+^ when treated with 1 μM LCTB-21, indicating that LCTB-21-treated NPCs do not progress through the cell cycle at the same rate as the NPCs treated with the DMSO vehicle (Fig. 2E). These data suggest that LCTB-21 treatment reduces the number of progenitors progressing through the cell cycle, likely due to cells arresting in the G_1_ phase as a result of DYRK1A inhibition and the imbalance in cyclin D1 and pT286-cyclin D1 levels.

To determine how quickly LCTB-21 and the reduction in DYRK1A activity affect cyclin D1 phosphorylation, we assessed pT286-cyclin D1 levels relative to total cyclin D1 levels over time in NPCs treated with 1 μM LCTB-21 (Fig. 2F). pT286-cyclin D1 was reduced in control and T21 NPCs within 1 h of treatment with LCTB-21, and this reduction persisted for at least 6 h (Fig. S1G). Concomitant with the decrease in T286 phosphorylation, cyclin D1 accumulated between 12 and 24 h post LCTB-21 treatment (Fig. S1H). Overall, inhibition of DYRK1A by LCTB-21 resulted in reduction of the ratio of pT286-cyclinD1 to total cyclin D1, which was maintained in a similar manner from 1 h to 24 h (Fig. 2F).

### LCTB-21 inhibits DYRK1A activity in human iPSC-derived cortical neurons in a dose-dependent manner

**Fig. 3.**
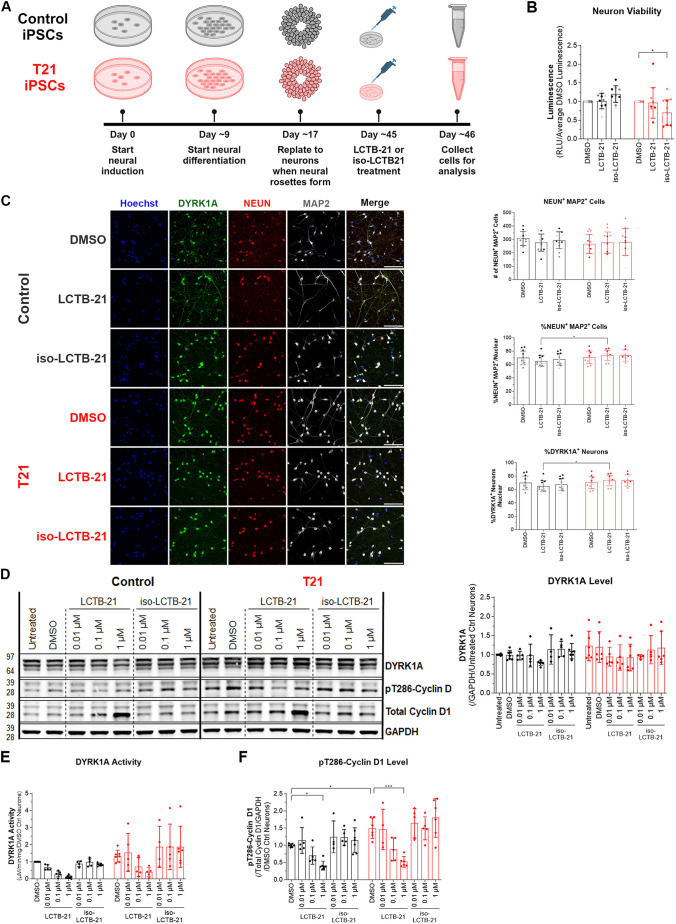
**LCTB-21 inhibits DYRK1A activity in human iPSC-derived cortical neurons in a dose-dependent manner.** (A) Schematic of iPSC-derived cortical neuron differentiation and treatment. NFT, neurofibrillary tangle. Created in BioRender by Bhattacharyya, A. (2026). https://BioRender.com/qmvb2l1 This figure was sublicensed under CC-BY 4.0 terms. (B) Viability quantification of control and T21 neurons treated with LCTB-21 and iso-LCTB-21 (*n*=3). LCTB-21 treatment has no effect on viability of 4-week cortical neurons compared to that of respective DMSO-treated neurons. (C) Representative images (40× objective; scale bars: 100 μm) and quantification of cortical neuron cultures (*n*=3). LCTB-21 treatment has no effect on the percentage of cortical neurons compared to that of respective DMSO-treated neurons. With LCTB-21 treatment, there is a slight increase in the number of T21 cortical neurons compared to controls. (D) Western blot and quantification of DYRK1A levels in control and T21 cortical neurons (control untreated, DMSO and 1 μM iso-LCTB-21 *n*=7; control, 0.01 μM LCTB-21, 0.1 μM LCTB-21 and 0.1 μM iso-LCTB-21 *n*=5; control 1 μM LCTB-21 *n*=6; control 0.01 iso-LCTB-21 *n*=4; T21 untreated, DMSO and 1 μM iso-LCTB-21 *n*=6; T21 0.01 μM LCTB-21, 0.1 μM LCTB-21 and 0.1 μM iso-LCTB-21 *n*=5; T21 0.01 iso-LCTB-21 *n*=4; T21 1 μM iso-LCTB-21 *n*=7). DYRK1A levels are the same in T21 and control neurons. Treatment with LCTB-21 does not affect the levels of DYRK1A. (E) Quantification of DYRK1A activity in cortical neurons (control DMSO, 1 μM LCTB-21 and 1 μM iso-LCTB-21 *n*=7; control 0.01 μM LCTB-21, 0.1 μM LCTB-21 and 0.1 μM iso-LCTB-21 *n*=5; control 0.01 iso-LCTB-21 *n*=4; T21 DMSO *n*=7; T21 0.01 μM LCTB-21, 0.1 μM LCTB-21, 1 μM LCTB-21 and 0.1 μM iso-LCTB-21 *n*=5; T21 0.01 iso-LCTB-21 *n*=4; T21 1 μM iso-LCTB-21 *n*=6). DYRK1A activity is increased in T21 cortical neurons. DYRK1A activity is decreased in a dose-dependent manner in control and T21 cortical neurons when treated with increasing concentrations of LCTB-21. (F) Western blot quantification of pT286-cyclin D1 relative to cyclin D1 levels in control and T21 cortical neurons (control DMSO, 1 μM LCTB-21 and 1 μM iso-LCTB-21 *n*=7; control 0.01 μM LCTB-21, 0.1 μM LCTB-21 and 0.1 μM iso-LCTB-21 *n*=5; control 0.01 iso-LCTB-21 *n*=4; T21 DMSO, 1 μM LCTB-21 and 1 μM iso-LCTB-21 *n*=6; T21 0.01 μM LCTB-21 *n*=4; T21 0.1 μM LCTB-21, 0.01 μM iso-LCTB-21 and 0.1 μM iso-LCTB-21 *n*=5). pT286-cyclin D1 levels decrease in a dose-dependent manner with LCTB-21 treatment. pT286-cyclin D1 levels in T21 neurons are higher than those in control neurons. pT286-cyclin D1 levels decrease in T21 and control neurons treated with 1 μM LCTB-21 relative to their respective DMSO-treated neurons. Results are from at least three technical replicates from separate stem cell differentiations (batches). The sample size (*n*) is defined by the number of batches. Data are presented as mean±s.d. *P*-values from two-way ANOVA with post-hoc tests are presented as *P≤0.05, ***P≤0.001. Two-way ANOVA results, post-hoc tests, *P*-values and excluded outliers are listed in Table S3.

Incorrect *DYRK1A* dosage is associated with intellectual disability (Duchon and Herault, 2016; Courraud et al., 2021; Ji et al., 2015); thus, it is important to understand how DYRK1A inhibition affects neuronal function. We tested the efficacy of LCTB-21 in iPSC-derived cortical neurons. NPCs were differentiated into TBR1^+^ cortical neurons (Fig. S2A) and treated with DMSO, LCTB-21 or iso-LCTB-21 (Fig. 3A). There was no difference in cell viability or DYRK1A activity in neurons treated with DMSO compared to that in untreated control neurons (Fig. S2B). A 24-h treatment with 1 μM LCTB-21 had no effect on the viability of control and T21 cortical neurons relative to that in those treated with DMSO, but there was an unexpected decrease in the viability of T21 cortical neurons treated with 1 μM iso-LCTB-21 (Fig. 3B).

Control and T21 iPSC-derived cultures had similar proportions of neurons (Fig. 3C). Treatment with LCTB-21 had no effect on the neuron composition relative to that with DMSO treatment (Fig. 3C). The T21 cultures treated with LCTB-21 had a slightly higher percentage of neurons than did control cultures (Fig. 3C). Cultures contained ∼70% NEUN^+^ (also known as RBFOX3^+^) MAP2^+^ neurons, and all neurons expressed DYRK1A (Fig. 3C).

DYRK1A protein levels were not significantly higher in T21 cortical neurons than those in controls (Fig. 3D). Nevertheless, consistent with the NPC data, DYRK1A activity was higher in T21 neurons than in controls (Fig. 3E). There was a dose-dependent decrease in DYRK1A activity following LCTB-21 treatment (Fig. 3E) in both control and T21 neurons. In postmitotic neurons, cyclin D1 plays a role in neuronal signaling and survival (Pedraza et al., 2023; Sumrejkanchanakij et al., 2003). pT286-cyclin D1 was higher in T21 cortical neurons than in control cortical neurons (Fig. 3F). Consistent with the decrease in DYRK1A activity with LCTB-21 treatment, pT286-cyclin D1 was decreased in a dose-dependent manner in T21 and control neurons treated with LCTB-21 (Fig. 3F). Thus, LCTB-21 reduces DYRK1A activity in a dose-dependent manner in iPSC-derived cortical neurons, whereas iso-LCTB-21 has no effect.

### DYRK1A inhibition decreases phosphorylation of the DYRK1A target, Tau, in human iPSC-derived cortical neurons

**Fig. 4.**
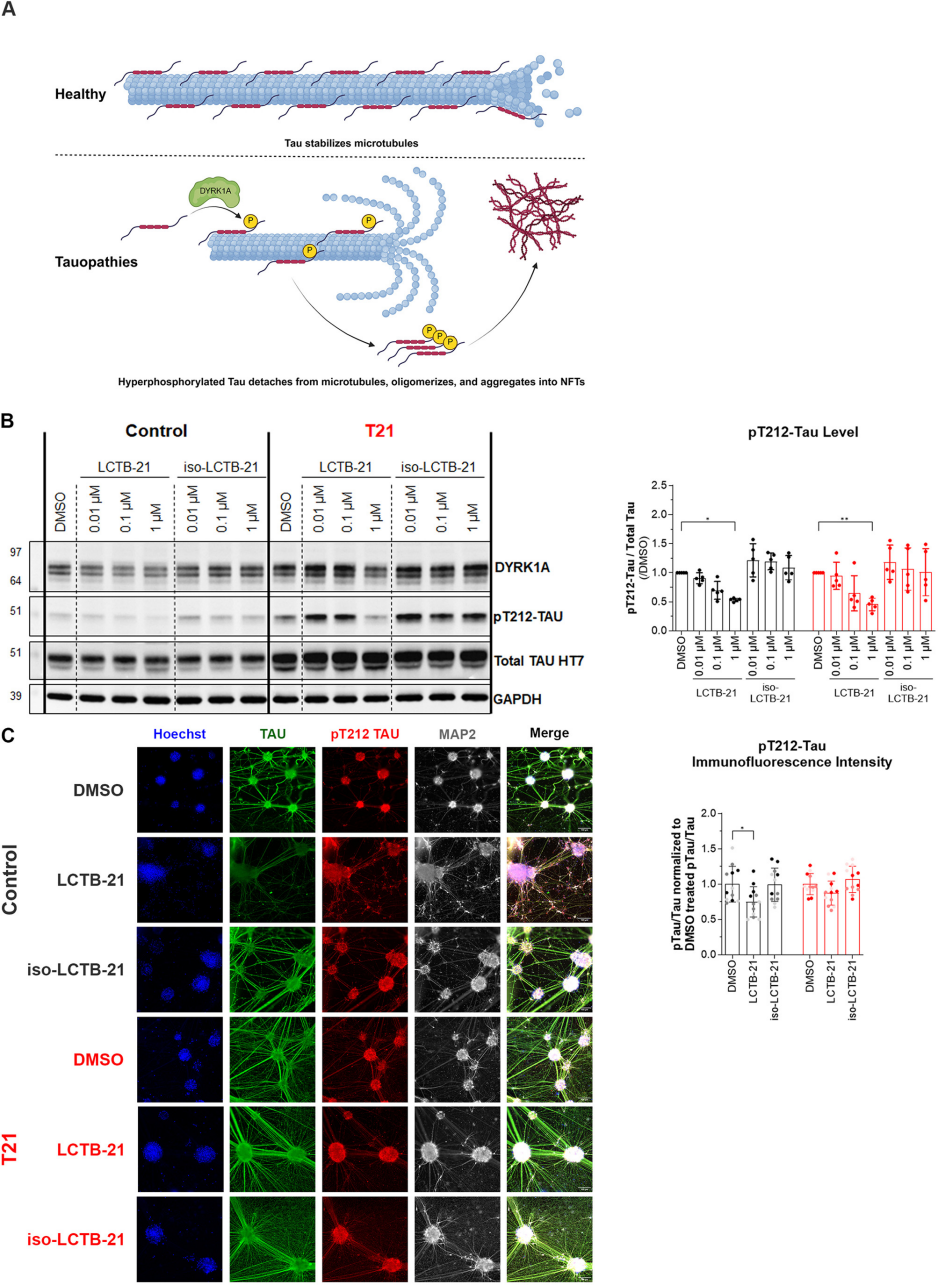
**DYRK1A inhibition decreases phosphorylation of the DYRK1A target, Tau, in human iPSC-derived cortical neurons.** (A) Schematic of DYRK1A and Tau phosphorylation. Created in BioRender by Bhattacharyya, A. (2026). https://BioRender.com/g2pg5y7. This figure was sublicensed under CC-BY 4.0 terms. (B) Western blot and quantification of total Tau and pT212 Tau levels in control and T21 cortical neurons (*n*=5). pT212 Tau levels decrease as DYRK1A activity decreases with increasing doses of LCTB-21. The same GAPDH control and total Tau (clone HT7) are shared with Fig. S2C as multiple targets were probed simultaneously on the membrane. (C) Representative images (20× objective; scale bars: 100 μm) and immunofluorescent intensity of pT212 Tau to total Tau normalized to the pT212 Tau/total Tau ratio of the respective DMSO-treated neurons (*n*=3). Results are from at least three technical replicates from separate stem cell differentiations (batches). The sample size (*n*) is defined by the number of batches. Data are presented as mean±s.d. *P*-values from two-way ANOVA with post-hoc tests are presented as *P≤0.05, **P≤0.01. Two-way ANOVA results, post-hoc tests, *P*-values and excluded outliers are listed in Table S3.

In neurons, DYRK1A phosphorylates diverse target proteins associated with differentiation and neuronal maintenance (Atas-Ozcan et al., 2021; Wang et al., 2017). The microtubule-associated protein, Tau (also known as MAPT), which helps maintain cellular structure and supports axonal transport (Tabeshmehr and Eftekharpour, 2023), is phosphorylated by DYRK1A at several sites, with Thr212 being the best characterized site – phosphorylation at this site primes for further phosphorylation by GSK3 at Ser208 (Ryoo et al., 2007; Kimura et al., 2007). Tau phosphorylation inhibits microtubule assembly and causes Tau to aggregate into neurofibrillary tangles (NFTs) (Parra Bravo et al., 2024; Aranda-Abreu et al., 2025) (Fig. 4A). Because LCTB-21 inhibited the activity of DYRK1A in iPSC-derived cortical neurons, we tested whether LCTB-21 reduces the phosphorylation of the DYRK1A target, Tau.

We found a dose-dependent decrease in Tau phosphorylated at Thr212 (pT212 Tau) relative to total Tau in 4-week iPSC-derived cortical neurons treated with LCTB-21 for 24 h (Fig. 4B). The inactive isomer iso-LCTB-21 did not affect levels of pT212 Tau (Fig. 4B). Consistent with the immunoblotting results, immunofluorescence intensity for pT212 Tau relative to total Tau decreased following treatment with 1 μM LCTB-21 (Fig. 4C). This reduction was significant in control neurons, while only a slight decrease in pT212 Tau/total Tau intensity was observed in the T21 neurons relative to that in in the respective DMSO-treated neurons.

LCTB-21 treatment also indirectly led to a decrease in pT217 Tau (Fig. S2C). DYRK1A-mediated phosphorylation of Tau primes Tau to be phosphorylated by other kinases at nearby residues (Liu et al., 2006; Cho and Johnson, 2003). As DYRK1A activity (Fig. 3E) and pT212 Tau levels (Fig. 4B) decreased with increasing dose of LCTB-21, pT217 Tau levels decreased in control and T21 neurons (Fig. S2C).

## DISCUSSION

*DYRK1A* is a dosage-sensitive kinase encoded on Hsa21 (Duchon and Herault, 2016). It is ubiquitously expressed across development in several tissues, including the brain (Atas-Ozcan et al., 2021; Guimera et al., 1999; Deboever et al., 2022; Becker and Joost, 1998; Hämmerle et al., 2008). DYRK1A regulates cellular processes in the brain such as cell cycle progression, neurogenesis, neuronal differentiation, axonal transport and synaptic plasticity (Yang et al., 2023; Cisternas et al., 2025). Dosage imbalance of *DYRK1A* and altered activity are associated with several neurodevelopmental disorders and neurodegenerative diseases (Deboever et al., 2022). *DYRK1A* haploinsufficiency causes the neurodevelopmental disorder DYRK1A syndrome, whereas DYRK1A overexpression contributes to DS pathology (Courraud et al., 2021; Deboever et al., 2022; Atas-Ozcan et al., 2021; Ji et al., 2015).

### LCTB-21 inhibits DYRK1A activity and reduces phosphorylation of DYRK1A targets

DYRK1A has been a target for pharmacological inhibition based on its role in synapse plasticity and neuron maintenance (Cisternas et al., 2025) and to mitigate the effects of *DYRK1A* dosage imbalance on learning and memory (Lindberg et al., 2023b; Stensen et al., 2021; Arbones et al., 2019; Deboever et al., 2022). Perha Pharmaceuticals' compound screens identified LCTB-21 as a potent, low-molecular-mass DYRK1A inhibitor that alleviates cognitive deficits including memory, learning and spatial recognition in mouse models of DS and AD (Deau et al., 2023; Lindberg et al., 2023a,b; Lindberg and Meijer, 2021). Although LCTB-21 has been shown to improve cognitive function in rodent models of DS and AD and is currently in a Phase 1 clinical trial, this is the first study to test LCTB-21 in a human cell model. This work demonstrates that LCTB-21 effectively inhibits DYRK1A activity in an iPSC-derived neural model of DS, establishing a platform for future investigations to delineate the mechanism of action of pharmacological DYRK1A inhibitors.

LCTB-21 reduces the activity of DYRK1A without affecting DYRK1A protein levels in human iPSC-derived NPCs. At baseline, DYRK1A activity is increased in T21 NPCs. The activity of DYRK1A decreases in a dose-dependent manner in control and T21 NPCs when treated with LCTB-21. DYRK1A activity is decreased within 1 h of LCTB-21 treatment, and the reduction persists for at least 24 h after treatment. The inactive isomer, iso-LCTB-21, has no effect on the activity of DYRK1A. Inhibition of DYRK1A activity via LCTB-21 has downstream effects in human NPCs, including decreased phosphorylation of cyclin D1 at Thr286.

When cyclin D1 is phosphorylated by DYRK1A, the transcription factor E2F remains bound and cannot bind to promoter regions, thus suppressing expression of cell cycle genes (Yang et al., 2006), including *MKI67* (encoding Ki-67). Increased expression of DYRK1A, thus, reduces the expression of cell cycle genes and, ultimately, proliferation by phosphorylating cyclin D1. pT286-cyclin D1 is reduced in both control and T21 cells upon LCTB-21 treatment and DYRK1A inhibition, concomitant with an increase in cycling Ki-67^+^ NPCs. The reduction of Ki-67^+^ T21 NPCs at the highest dose of LCTB-21 (Fig. 2D) is likely due to the toxicity observed in T21 NPCs with 1 μM LCTB-21 treatment (Fig. S1F). Consistent with our data, DYRK1A inhibition increases the number of cycling cardiomyocytes and β-cells (Young et al., 2022; Shen et al., 2015; Bertrand et al., 2025). Although we observe an increase in Ki-67^+^ NPCs with LCTB-21 treatment, the accumulation of cyclin D1 raises the possibility that Ki-67^+^ control and T21 NPCs are accumulating in the G_1_ phase. High cyclin D1 levels are required during G_1_ but decline as the cell progresses to S phase (Yang et al., 2006). The reduction in the number of pHH3^+^ NPCs indicates that fewer LCTB-21-treated cells reach late G_2_ and M phases, further supporting accumulation of cells in G_1_. DYRK1A-mediated cyclin D1 regulation could explain the cell cycle deficits reported in DS (Sharma et al., 2022; Contestabile et al., 2007; Najas et al., 2015); however, this work highlights the importance of timing and dosage of treatment to regulate cell cycle dynamics.

LCTB-21 reduces the catalytic activity of DYRK1A in human iPSC-derived cortical neurons. The activity of DYRK1A decreases in a dose-dependent manner in control and T21 neurons when treated with LCTB-21; however, the inactive isomer, iso-LCTB-21, has no effect on the activity of DYRK1A. The inhibition of DYRK1A activity in iPSC-derived cortical neurons by LCTB-21 causes a decrease in phosphorylation of downstream targets cyclin D1 at Thr286 and Tau at Thr212. The microtubule-associated protein, Tau, stabilizes microtubules, providing support for axonal transportation and cytoskeletal structure. Tau phosphorylation is a dynamic process allowing reorganization of the cytoskeleton. However, when there is an imbalance in this process and Tau becomes hyperphosphorylated, it cannot bind as readily to microtubules. Hyperphosphorylated Tau is prone to misfolding and aggregates into insoluble neurofibrillary tangles, which impairs neuron function (Boutajangout et al., 2011; Lasagna-Reeves et al., 2011).

Hyperphosphorylated Tau and neurofibrillary tangles are common pathological hallmarks in several neurodegenerative disease, including AD and DS-associated AD (DS-AD) (Zhang et al., 2024; Sheppard et al., 2012). In these diseases, Tau can be hyperphosphorylated at several sites, including Thr212 (Miao et al., 2019; Alonso et al., 2010), by kinases including DYRK1A (Ryoo et al., 2007). Consistent with other reports of DYRK1A inhibition reducing phosphorylation of Tau (Shukla et al., 2023; Chen et al., 2024; Tu et al., 2024; Frost et al., 2011), we show that treatment with a DYRK1A inhibitor, LCTB-21, reduces the phosphorylation of Tau at Thr212, which may indirectly lead to a reduction in the phosphorylation at Thr217 by other kinases. This reduction of pT212 Tau demonstrates the potential of LCTB-21 to limit Tau hyperphosphorylation and aggregation, supporting its therapeutic use for mitigating Tau pathology associated with AD, DS-AD and other tauopathies.

Taken together, our results demonstrate that LCTB-21 decreases DYRK1A activity in human T21 iPSC-derived NPCs and cortical neurons. Phosphorylation of DYRK1A targets is also reduced with LCTB-21 treatment. We show, for the first time, that LCTB-21 decreases DYRK1A dosage effects in a relevant human disease model, supporting future human trials.

### Implications for LCTB-21 treatment

Among other kinases, DYRK1A has emerged as a therapeutic target for neurodevelopmental disorders including DS, neurodegenerative diseases including AD and Parkinson's disease, cancers, diabetes and myocardial infarction (Radhakrishnan et al., 2016; Rammohan et al., 2022; Feki and Hibaoui, 2018; Murphy et al., 2024; Deboever et al., 2022; Wegiel et al., 2011). LCTB-21 has been shown to correct cognitive deficits in mouse models of DS (Lindberg et al., 2023b), and the present study reports the efficacy of LCTB-21 in human neural cells. Together, these data suggest that LCTB-21 could be a promising DYRK1A inhibitor used to help mitigate cognitive decline in individuals with DS as well as in those with other diseases associated with increased DYRK1A activity.

With the correct dosage and timing of treatment, LCTB-21 could potentially be used to reduce or slow AD pathology in DS, including the accumulation of NFTs and amyloid-beta (Aβ) plaques. Overexpression of DYRK1A causes hyperphosphorylation of Tau, which accumulates as insoluble NFTs and causes neuronal dysfunction and degeneration (Ryoo et al., 2007; Liu et al., 2008). DYRK1A-mediated reduction of Tau hyperphosphorylation could delay neuron dysfunction and degeneration and, ultimately, slow cognitive decline in DS and other tauopathies. DYRK1A has also been shown to affect the processing of amyloid precursor protein (APP) and Aβ plaques. Increased expression of DYRK1A increases the phosphorylation of APP at Thr668 (Ryoo et al., 2008). This phosphorylation promotes preferential binding of β-secretase to APP, leading to increased cleavage and Aβ peptide production, whereas reduced Thr668 phosphorylation is associated with lower Aβ accumulation (Ryoo et al., 2008; Lee et al., 2003). The knockdown of DYRK1A rescues cognitive impairment and decreases Aβ and Tau pathology in the 5xFAD mouse model of AD (Lee et al., 2025). Reducing the phosphorylation of APP at Thr668 by inhibiting DYRK1A could reduce the amyloidogenic cleavage of APP by β-secretase and could slow the formation of Aβ plaques in DS. Because of the similarities in the clinical progression of DS-AD, early-onset or familial AD, and late-onset or sporadic AD (Fortea et al., 2021; Rafii, 2020; Strydom et al., 2018; Gomez et al., 2020), LCTB-21 may also serve as a promising therapeutic to delay NFT and Aβ accumulation in familial AD and sporadic AD. It would also be of interest to determine whether reduction of DYRK1A activity using LCTB-21 rescues alterations in cytoskeletal and synaptic vesicle protein networks disrupted in T21 neurons, as observed with DYRK1A copy number rescue (Wu et al., 2022).

### Limitations of the study

A limitation of this study is that only one pair of T21 and control iPSC-derived cell lines were used to test the effects of LCTB-21. It is critical to repeat the experiments in more cell lines to address individual genetic variability and provide broader impact. We mitigated this issue to some extent by using an isogenic pair of iPSCs (Giffin-Rao et al., 2022). Isogenic cell lines are a powerful model to study disorders and diseases because the controls have the same genetic background as the disease model. Future studies will need to test the efficacy of LCTB-21 in more biological replicates to confirm the results presented in this study.

The goal of this study was to determine whether LCTB-21 is able to inhibit DYRK1A activity and, consequently, reduce the phosphorylation of downstream targets in human cells. DYRK1A activity is inhibited in mouse HT-22 hippocampal neuronal cells when treated with LCTB-21 for 4 h (Lindberg et al., 2023b), so to first confirm that DYRK1A is inhibited in human cells with LCTB-21, we analyzed the inhibition over a 24-h time period. Additional studies with prolonged LCTB-21 treatment are needed to address the long-term effects of LCTB-21 treatment and DYRK1A inhibition and to delineate the mechanism of action in human cells.

Additional studies with other DYRK1A inhibitors or other genetic tools to manipulate DYRK1A expression are needed to validate that the data presented here are indeed a result of DYRK1A inhibition. Given the high similarity in the catalytic domains of kinases, most DYRK1A inhibitors also target DYRK1B and CDC2-like kinases (CLKs) (Lindberg et al., 2023a; Lindberg and Meijer, 2021), making it challenging to attribute specific effects to a single kinase. However, a comparative analysis of over 50 DYRK/CLK inhibitors demonstrated that all compounds active against DYRK1A consistently affect Tau phosphorylation at T212 (Lindberg et al., 2023a). Overexpression and knockout of *DYRK1A* and inhibition of DYRK1A consistently affect the levels of cyclin D1 (Soppa et al., 2014; Recasens et al., 2021; Laham et al., 2024; Hille et al., 2016; Chen et al., 2013). The DYRK1A-mediated consistency in Tau phosphorylation and cyclin D1 levels across multiple studies suggests that the observations presented here are likely due to DYRK1A inhibition. This work confirms that LCTB-21 inhibits DYRK1A activity and phosphorylation of downstream targets in a T21 iPSC-derived neural model. Nevertheless, because immobilized LCTB-21 was recently reported to bind, directly or indirectly, to hundreds of proteins in the rat brain (Deau et al., 2026), a comprehensive characterization and comparison of protein targets in both disomic and trisomic iPSC-derived NPCs and neurons would provide deeper insight into its mechanism of action.

Our data confirm that LCTB-21 decreases the kinase activity of DYRK1A and specifically decreases phosphorylation of a critical target, Tau. The long-term and physiological effects of reduced DYRK1A activity, namely the ability to rescue neuronal defects in T21, are important future considerations.

## MATERIALS AND METHODS

### Drug synthesis

Leucettinib-21 (LCTB-21) and iso-Leucettinib-21 (iso-LCTB-21) were developed and synthetized by Perha Pharmaceuticals as previously described (Lindberg et al., 2023b; Deau et al., 2023).

### Statistical analysis

Results are from at least three technical replicates from separate stem cell differentiations (batches). The sample size (*n*) is defined by the number of batches and is denoted in each figure legend. Data were analyzed with two-way ANOVAs and appropriate post-hoc corrections in GraphPad Prism. Data are presented as mean±s.d. *P*-values from two-way ANOVA with post-hoc tests are presented as *P≤0.05, **P≤0.01, ***P≤0.001, ****P≤0.0001. Outliers were identified with Grubbs' test (α=0.01) in Prism and excluded from the analysis. Two-way ANOVA results, post-hoc tests, *P*-values and excluded outliers are listed in Table S3.

### Cell culture

Isogenic control (WC-24-02-DS-B) and T21 (WC-24-02-DS-M) iPSCs were established in the Bhattacharyya laboratory and are available at WiCell (Madison, WI, USA; Giffin-Rao et al., 2022). We verified cell quality by mycoplasma testing performed routinely throughout the duration of experiments and karyotyping of each line at the conclusion of experiments to ensure that karyotypes remained as expected (WiCell). iPSCs were cultured at 37°C/5% CO_2_ in six-well plates on mouse embryonic fibroblasts (MEFs; WiCell) with daily changes of human embryonic stem cell medium (Table S1). iPSCs were passaged weekly onto fresh MEFs using 1 mg/ml Collagenase Type IV (Gibco) in Dulbecco's modified Eagle medium/F12 (Gibco). At the start of neural induction on Day 0, iPSCs were treated with 10 μM SB431542 (Peprotech), 0.1 μM LDN-193189 2HCl (Selleckchem) and 2 μM XAV-939 (TOCRIS) in neural induction medium. At Day 8 or 9, cells were replated at a 1:1 ratio from MEFs to six-well Matrigel-coated plates. Cells were dissociated with TrypLE Express (Gibco) at 37°C for 5 min. TrypLE Express was removed, and 2 ml Neurobasal (Gibco) was added to each well. Cells were gently removed from the well by pipetting. Cells were transferred to a 15 ml tube and centrifuged at 300 ***g*** for 2 min. Cells were resuspended in 2 ml/well NPC medium (Table S1) with 5 μM Y-27632 dihydrochloride (TOCRIS). NPC medium was changed daily.

### Treatment of iPSC-derived NPCs

Once NPCs formed neural rosettes, they were treated with DMSO, LCTB-21 or iso-LCTB-21 in NPC medium (Table S1) for the indicated time. For western blot and kinase activity analysis, medium was removed, and 1× Dulbecco's PBS (dPBS) was added to each well. Cells were scraped off the well and transferred to an Eppendorf tube. Cells were pelleted at 4500 rpm for 4 min, supernatant was removed, and cell pellets were stored at −80°C until analysis. For immunocytochemistry, medium was removed, and cells were fixed in 4% paraformaldehyde (PFA) for 15 min. NPCs were washed and stored in 1× PBS until analysis.

### Treatment of iPSC-derived cortical neurons

Once NPCs formed neural rosettes (Hunt et al., 2019; Fedorova et al., 2019), cells were dissociated with Accutase (Millipore Sigma) at 37°C for 5 min. Single cells were transferred to a 15 ml tube and centrifuged at 300 ***g*** for 2 min. Supernatant was removed, and cells were resuspended in NPC medium. Cells were plated with 5 μM Y-27632 dihydrochloride and 200 nM γ-Secretase Inhibitor XXI, Compound E (Millipore Sigma). The following day, half of the medium was replaced with neural differentiation medium (NDM) (Table S1) with 200 nM γ-Secretase Inhibitor XXI, Compound E. Half-media changes were done every 3–4 days. 4 weeks after plating, cortical neurons were treated with DMSO, LCTB-21 or iso-LCTB-21, in NDM for 24 h. For western blot and kinase activity analysis, medium was removed, and 1× dPBS was added to each well. Cells were scraped off the well and transferred to an Eppendorf tube. Cells were pelleted and stored at −80°C until analysis. For immunocytochemistry, medium was removed, and cells were fixed in 4% PFA for 15 min. Neurons were washed and stored in 1× PBS until analysis.

### Cell viability assay

NPCs and neurons were plated at a density of 50,000 cells per well in a 96-well plate. 24 h after plating, cells were treated with DMSO, LCTB-21 or iso-LCTB-21. 24 h after treatment, medium was replaced, and the CellTiter-Glo^®^ 2.0 Assay (Promega) was performed according to the manufacturer's protocol. Luminescence was recorded with a Promega GlomMax Multi Detection System.

### Western blotting

Cell pellets were lysed on ice in homogenization buffer [25 mM MOPS (Sigma-Aldrich), 15 mM EGTA (Sigma-Aldrich), 15 mM MgCl_2_ (Sigma-Aldrich), 60 mM β-glycerophosphate (Sigma-Aldrich), 15 mM p-nitrophenylphosphate (Sigma-Aldrich), 2 mM DL-dithiothreitol (DTT; Sigma-Aldrich), 1 mM Na_3_VO_4_ (Sigma-Aldrich), 1 mM NaF (Sigma-Aldrich), 1 mM di-sodium phenylphosphate (Sigma-Aldrich), protease inhibitor cocktail (Complete, Roche), pH 7.2] supplemented with 0.1% Nonidet P-40, and then centrifuged (17,000 ***g*** for 1 min at 4°C). Protein extracts were mixed (1:1 v/v) with sample buffer (2× NuPAGE LDS sample buffer, 200 mM DTT). Following heat denaturation, 25 μg of proteins were loaded on NuPAGE precast 4-12% Bis-Tris protein gels (Thermo Fisher Scientific). Electrophoresis was performed in MOPS buffer. Rapid blot transfers were carried out at 2.5 A/25 V for 7 min. Membranes were blocked in milk [5% Regilait in Tris-buffered saline with 0.1% Tween 20 (TBST)] for 1 h. Membranes were then incubated with primary antibodies (Table S2). Finally, membranes were incubated for 1 h at room temperature with horseradish peroxidase-conjugated secondary antibodies (Table S2), and chemiluminescent detection was achieved with homemade ECL-Tris buffer (100 mM Tris-HCl pH 8.5, 0.009% H_2_O_2_, 0.225 mM *p*-coumaric acid, 1.25 mM luminol) with Fusion Fx7 camera software. Bands were quantified using ImageJ. Experiments were repeated at least three times.

Multiple targets were probed simultaneously on each membrane. The targets were divided between figures and share the same GAPDH control. GAPDH controls are shared between Fig. 1C and Fig. 2B, Fig. 1F and Fig. 2F, and Fig. 4B and Fig. S2C. pT212 Tau and pT217 Tau levels were normalized to total Tau HT7 and probed for on the same membrane. Total Tau HT7 is shared between Fig. 4B and Fig. S2C.

### Kinase activity assay

Analysis of DYRK1A activity was performed on cell lysates using high-performance liquid chromatography (HPLC) as previously described (Bui et al., 2014). To assess the activity of DYRK1A by HPLC, a Dansyl-conjugated peptide was designed, Dansyl–KKISGRLSPIMTEQ-NH_2_ (Dan–peptide), the sequence of which is derived from the human forkhead transcription factor FKHR (also known as FOXO1). This transcription factor is known to be phosphorylated by DYRK1A on the Ser329 residue of the GRLSPIM motif. This peptide and its Ser329 phosphorylated product should be easily separated by reverse-phase HPLC and specifically quantified by detection of the fluorescence of the Dansyl moiety. The purity and identity of the fluorescein-labeled peptide substrate (Dan–peptide) was initially assessed by reverse-phase HPLC [Prominence Shimadzu ultra-fast liquid chromatography (UFLC) system interfaced with LabSolutions software]. Samples were injected into a Nucleodur C18 column (length, 150 mm; internal diameter, 4.6 mm; particle size, 5 μm) at 45°C. The mobile phase used for the separation consisted of two eluents; solvent A was water with 0.1% trifluoroacetic acid (TFA), and solvent B was acetonitrile with 0.1% TFA. Compounds were separated by an isocratic flow (85% A/15% B) rate of 1.0 ml/min. The products were monitored by fluorescence emission at 537 nm after excitation at 375 nm and quantified by integration of the peak absorbance area, employing a calibration curve established with various known concentrations of peptides. Incubation of DYRK1A with the Dan–peptide in the presence of ATP leads to two peaks corresponding to the FAM–peptide (retention time of 5.5 min) and its phosphorylated form (retention time of 4.8 min). As expected, the amount of phosphorylated Dan–peptide calculated by integration of the peak (area under the curve) increases linearly with the time of incubation. Assays were performed in a 96-well enzyme-linked immunosorbent assay plate in a total volume of 50 μl consisting of kinase buffer (50 mM Tris-HCl, 10 mM DTT and 5 mM MgCl_2_), ATP (up to 1000 μM), Dan–peptide substrate (up to 100 μM), and purified DYRK1A–DC (up to 0.5 ng) or cell extracts (25 μg total protein extract). Briefly, samples containing the enzyme were preincubated with peptide substrate at 37°C for 1 min, and the reaction was started by the addition of ATP. At different time points (up to 30 min), 50 μl HClO_4_ (15% in water) was added to stop the reaction, and 20 μl was automatically injected into the HPLC column.

### Immunocytochemistry

After treatment, cells were fixed in 4% PFA for 15 min. Cells were blocked with 5% normal donkey serum (NDS) and permeabilized with 0.2% TX-100 in 1× PBS for 15 min at room temperature. Primary antibodies at desired concentrations (Table S2) with 5% NDS in 1× PBS were applied overnight at 4°C. Primary antibody solutions were washed off with 1× PBS. Secondary antibodies at 1:500 with 5% NDS in 1× PBS were applied for 30 min at room temperature. Cells were washed with 1× PBS and stained with 1:100 Hoechst 33342 for 5 min at room temperature. Cells were washed with 1× PBS and mounted with Fluoromount-G™ (Invitrogen).

### High-content imaging analysis

NPCs and neurons were plated on 96-well plates with #1.5 bottom (Cellvis). Proliferation and cell culture composition analyses were carried out with a Nano High-Content Imaging System (Molecular Devices) with the MetaXpress software. Four to six wells per treatment condition were acquired. NPC images were acquired at 20× with nine sites/well. Neuron images were acquired at 20× with 16 sites/well. Representative images were acquired at 20× or 40× using a Nikon Ti2 Eclipse microscope.

## Supplementary Material

10.1242/dmm.052740_sup1 Supplementary information

Table S3. Statistical analysis summary.
